# Robustness of Multi-Project Knowledge Collaboration Network in Open Source Community

**DOI:** 10.3390/e25010108

**Published:** 2023-01-04

**Authors:** Xiaodong Zhang, Shaojuan Lei, Jiazheng Sun, Weijie Kou

**Affiliations:** 1 School of Economics and Management, University of Science and Technology Beijing, Beijing 100083, China; 2 Nuclear and Radiation Safety Center, Beijing 100082, China

**Keywords:** open source community, multi-project collaboration, directed weighted network, robustness

## Abstract

Multi-project parallelism is an important feature of open source communities (OSCs), and multi-project collaboration among users is a favorable condition for an OSC’s development. This paper studies the robustness of this type of community. Based on the characteristics of knowledge collaboration behavior and the large amount of semantic content generated from user collaboration in open source projects, we construct a directed, weighted, semantic-based multi-project knowledge collaboration network. Using analysis of the KCN’s structure and user attributes, nodes are divided into knowledge collaboration nodes and knowledge dissemination nodes that participate in either multi- or single-project collaboration. From the perspectives of user churn and behavior degradation, two types of failure modes are constructed: node failure and edge failure. Based on empirical data from the Local Motors open source vehicle design community, we then carry out a dynamic robustness analysis experiment. Our results show that the robustness of our constructed network varies for different failure modes and different node types: the network has (1) a high robustness to random failure and a low robustness to deliberate failure, (2) a high robustness to edge failure and a low robustness to node failure, and (3) a high robustness to the failure of single-project nodes (or their edges) and a low robustness to the failure of multi-project nodes (or their edges). These findings can be used to provide a more comprehensive and targeted management reference, promoting the efficient development of OSCs.

## 1. Introduction

As the physical carrier for the implementation of open source modes and ideas, the open source community (OSC) has accumulated many volunteers from all over the world. Through in-depth interaction and collaboration of volunteers, OSC members have broken through the barriers of traditional production methods in a creative storm, collaborative design, knowledge sharing, and dissemination. The OSC’s many advantages (e.g., openness, low running cost, high efficiency, high project conversion rate, and high customer satisfaction) have made it highly valued by people from all walks of life, as well as by governments, enterprises, and universities around the world [1,2,3].

The open source model was first applied to open source software (i.e., freeware) [4,5,6], where its many advantages (e.g., cost efficiency, low risk of trial and error, fostering talent, and other aspects) have led to 97% of individual developers and 99% of enterprises now using such software worldwide [7]. Under the philosophy that all knowledge should be freely available to everyone, the open source model has since established itself in many specific industries (e.g., industrial design, e-commerce, education, commercial marketing), and many product users have gone on to become product developers through community collaboration [8,9,10,11]. However, despite many successful OSCs and fruitful academic research results, there are also many cases of failure. For example, regarding the OSCs built by most of the Fortune 100 companies, their expected returns in product innovation and economic benefit have not been achieved because of low user collaboration, lack of valuable contributions, serious loss of user resources and other factors [12,13]. The openness, knowledge characteristics, democracy, and collaborative nature of the OSC itself determine that any rigid organizational structure or strong organizational control will inhibit the creativity of the community, which will ultimately lead to the loss of users and the failure of open source projects (OSPs). Therefore, it is of great practical significance for the stable development of OSCs and OSPs to (1) study the evolution process of the KCN’s structure and user attributes, and (2) build a multi-project knowledge collaboration network (KCN) and study its robustness and failure conditions.

The robustness of a network is the ability to maintain the structural integrity and functional continuity of the system in case of failures of nodes or links [14,15,16]. Many scholars have researched the robustness of the OSC, providing practical and valuable insights. However, some issues remain in the areas of network construction and attack strategy.

First, regarding network construction, whether through empirical and qualitative research methods or simulation modeling and experimental analysis, many studies build their social network models using social network methods and system analysis ideas from complex networks. For example, Zhang et al. [17] built an undirected, unweighted network based on the knowledge collaboration between users to study the topology characteristics and robustness of the open IDEO open source design network. Knowledge collaboration distinguishes OSCs from other online communities, and constructed networks should reflect this. Zhou et al. [18,19,20] built a KCN to study the impact of user loss in the OSP community on the robustness of OSPs and OSCs. They screened out behaviors where users were in deep knowledge collaboration with each other. However, their constructed KCN does not reflect the content of knowledge collaboration, that is, the strength of knowledge collaboration is reflected only by the frequency of collaboration, which is not fully realistic. When building a semantic-based KCN, Lei et al. [21,22] considered the frequency and content strength of knowledge collaboration between users, but their research was limited to individual OSPs in the OSC, not to the multi-project OSC as a whole.

Second, regarding attack strategies, there are usually two types of attacks: random attacks and targeted attacks [23,24], where targeted attack strategies are mainly based on network topology information [25]. Basing the attack strategy on network topology information was first proposed by Holme et al. [26] when measuring the robustness of scientific cooperation networks and Internet traffic networks. They considered the deletion of nodes and edges based on degrees and betweenness, respectively. Later, many scholars studied the robustness of various complex networks based on the combination and expansion of the above attack strategies. For example, Bellinger et al. [27] designed (a) node failure, which is based on the order of node degree, node strength, node intermediary, and weighted node intermediary, and (b) edge failure, which is based on the order of edge weight removal from strong to small and weak to large. They constructed the respective unweighted and weighted networks of six real networks (e.g., the American airport flight transportation network, and British teachers’ social network) to study how the difference in weight affects them. Iyer et al. [28] also conducted comparative experiments in six real networks, using not only node removal strategies of node degree and node betweenness but also the change rule of network connectivity under the strategy of removing nodes by eigenvector centrality and closeness centrality. Zhang et al. [29] studied methods to improve the robustness of small-world networks, deleting nodes or edges in order of their edge weight, edge betweenness, and node betweenness. Their results show that as the average degree of the network increases, so does the robustness of the network. Tang et al. [30] adopted a node removal strategy based on the node betweenness in their research on the robustness of the regional collaborative innovation network structure. Based on the analysis of the above literature, we found that the attack strategy is mainly based on the network topology information, that is, the structural characteristics of nodes or edges. However, multi-project parallelism is an important feature of OSCs. Research shows that developers participate in multiple OSPs at the same time and are willing to make long-term contributions. They focus on one or two active projects at a time, and after finishing their activity in a project, they soon select a new project in which to collaborate [31]. The obvious project characteristics of community users are rarely noticed in most robustness studies.

Further, most attack strategies are based on node failure. Open source design is an innovative design mode, with core characteristics that include self-organization and large-scale, deep collaboration of its users. Many studies have pointed out that collaborative behavior degradation and failure (i.e., edge degradation and failure) have a serious impact on the network [32,33]. For example, Griffith et al. [34] stated that the collaboration network formed by large-scale interactive discussion and collaboration among users is the best way to share, innovate and disseminate knowledge. Singh et al. [35] asserted that the deep collaboration behavior represented by mutual comments among users in the OSC is the power source that enables new products to be quickly recognized and distributed, and effectively improves the efficiency of product development. Using user activity and project attraction as indicators. Midha et al. [36] studied the factors that affect the success of the OSC. They found that attracting many users into the community and guiding users to continue their interaction and collaboration are the fundamental elements for the survival and development of the OSC. Many community failures are caused by users leaving or having a reduced willingness to collaborate. Li et al. [37] stated that the autonomy and mobility of community participants have an important impact on the network structure and sustainable development of the community. Crowston et al. [38] argued that the quality and quantity of user collaboration are more important than the number of users entering the community. Ransbotham et al. [39] stated that a very important reason for the inefficient operation of an OSC is the lack of truly valuable contributions by community members. These studies show that user mobility and collaboration behavior are key to the success of the community or project. In this robustness study, we comprehensively consider the impact of node and edge failures on network performance, so that more targeted management strategies can be formulated for the efficient and stable development of the OSC.

The remainder of this paper is organized as follows. In Section 2, we construct a directed weighted KCN that takes into account the characteristics of multi-project collaboration in the open source design community and the large amount of semantic text information generated in the user collaboration process. We also analyze the KCN’s structure and user attributes, dividing nodes into knowledge collaboration nodes and knowledge dissemination nodes that participate in either multiple- or single-project collaboration. In Section 3, two basic failure modes (i.e., node failure and edge failure) are combined to construct the robustness research framework. In Section 4, a simulation model is built to determine changes in network performance for each of the failure modes. Our conclusions are given in Section 5, including our recommendations for community management strategies.

## 2. Construction and Characteristics Analysis of Multi-Project KCN

### 2.1. Construction of Semantic-Based KCN

#### 2.1.1. Data Selection and Processing of Empirical Research

We use the project data of Local Motors (an open source automobile community) as the research object. Until its dissolution in 2022, Local Motors was the largest car design exchange community in the world, with nearly 10,000 care enthusiasts from 121 countries. They had an online community for designing and communicating automobile-related products and a microfactory for their offline production and manufacturing. Their community’s innovation process, project design process, research and development, and production process reflect typical characteristics of an OSC, such as a high concentration of users and project types, the strong creation and dissemination of knowledge, and the closed-loop integration of production and marketing. They, therefore, make a suitable research object for this study. Specifically, we select 11 projects from the Local Motors OSC as research objects: 3D-PC, Airbus C, Air C, Darpa, LM SF-01, Olli, Open T, Rally F, Road R, Sketchover, and Verrado DT. We crawl the complete collaboration data, which includes project information, time information, and comments. According to the project statistics, 1689 users participated in 11 project collaborations, with a total of 25,472 collaborative interactions.

**Figure 1:**
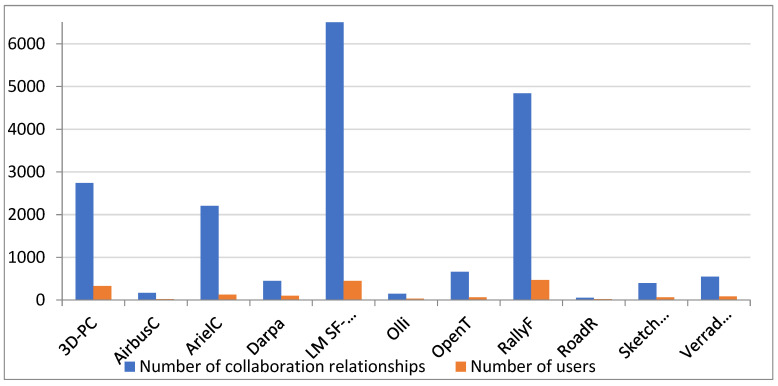
Number of users and knowledge collaboration interactions in each project.

In an OSC, users participate in product design through large-scale, in-depth interaction and collaboration, where “knowledge collaboration” is the main means with which to achieve innovative design. Therefore, it is necessary to establish a KCN rather than an information dissemination network. The first step when building a KCN is to screen for interactions that have knowledge collaboration characteristics. We use machine learning to screen out the knowledge collaboration behaviors from all communication behaviors. As shown in Figure 1, between 20 May 2008 and 15 November 2018, there were 18,469 knowledge collaboration interactions throughout the 11 Local Motors OSPs, which were completed by 1410 users.

#### 2.1.2. Construction Method of Semantic-Based Multi-Project KCN

To successfully build the KCN of an OSC, an in-depth analysis of knowledge collaboration behavior is essential. Knowledge collaboration behavior includes both (a) knowledge-level behavior, which is measured by the collaborative content between users, and (b) non-knowledge-level behavior, which is measured by the collaborative frequency. However, most research on OSC networks considers only the collaborative frequency (i.e., the non-knowledge-level behavior) between users when calculating the network weight. If the collaborative content (i.e., the knowledge-level behavior) between users is not also considered, then the intensity of the collaboration between users is not truly reflected.

To construct a directed, weighted, semantic-based KCN, $G\left(V,\;E,\;W\right)$: (1) the knowledge collaboration users and knowledge dissemination users are taken as the nodes, V; (2) the collaboration behaviors between nodes are taken as the connecting edges, E; and (3) the weighted processing of collaboration content and collaboration frequency (i.e., both parts of knowledge collaboration behavior) by users are taken as the edge weight, W.

**Figure 2:**
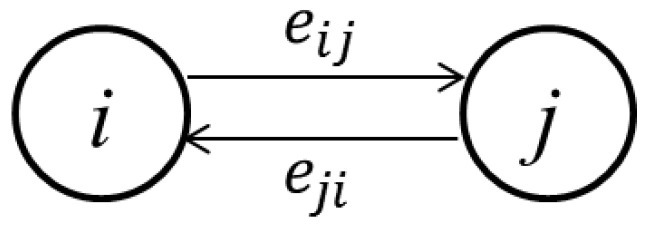
Schematic diagram of network nodes and connections.

As shown in Figure 2, users are taken as nodes, $v_{i}$, and the collaboration behaviors between nodes are taken as edges, $e_{ij}$. The edge weight, $\omega_{ij}$, is obtained by weighting the collaborative content intensity, $g_{ij}$, and the collaborative frequency intensity, $k_{ij}$, between users. The calculation of the edge weight of the network is:

(1) $$\left\{\begin{array}{cc} \omega_{ij}=\alpha g_{ij}+\beta k_{ij} & k_{ij}>0\\ 0 & k_{ij}<0\; \end{array}\right.$$

where α and β are the influence coefficients of the content intensity and frequency intensity, respectively, satisfying α+β=1. The collaborative frequency intensity, $k_{ij}$, is obtained by normalizing the one-way collaboration times, $k_{ij}$, from designers $\;v_{i}$ to $\;v_{j}$.

The collaborative content intensity, $g_{ij}$, is calculated by the matching degree of keywords contained in user comments. The keyword matching score calculated from a user’s comments on the whole project or community is the semantic weight of the user, $g_{i}$. Here, the calculation of content intensity, $\;g_{ij}$, includes word segmentation, construction of the co-occurrence matrix and calculation of the candidate word weight, among others, as described in detail by Lei [21,22].

### 2.2. Analysis of User Behavior Characteristics and Structural Attributes of the Network

To study a network’s robustness, it is a prerequisite to deeply analyze the network characteristics and the formation process of collaborative behavior. Specifically, for a KCN, individual user characteristics and behaviors should be considered.

#### 2.2.1. Structural Attributes of the Network

For the construction of a semantic-based, multi-project KCN, Ucinet software is used to measure the network size, some static topology parameters and the network characteristics, as shown in Table 1.

In Table 1, network size reflects the number of users involved in the network (i.e., the nodes) and the degree of collaboration between users (i.e., the edges). The average out-strength and density of the network are low; these topological parameters are calculated based on the directed weighted network, where the value of the edge weight is within the range of [0, 1], resulting in the small values shown. However, this also shows that users who enter the community have a greater potential for knowledge collaboration. It is necessary to analyze the knowledge collaboration behavior of different users and provide corresponding incentive strategies. The clustering coefficient is 0.097 and the average path length is 3.105, which indicates that the network shows obvious small-world characteristics and users in the network form multiple small groups of different sizes. There are key “connections” between small groups that connect the whole network. This is because of the open source nature of the community, that is, user autonomy enables them to choose which projects they collaborate on. Some users participate in many projects, while others only participate in those projects they find most interesting.

#### 2.2.2. Node Attributes

The high degree of autonomy of community members in the OSC determines that users can freely choose collaborative objects and collaborative projects. To comprehensively examine the characteristics of nodes, we analyze them from two perspectives: structural attributes and project attributes. The structural attributes of nodes include the position, role, and cooperation degree of nodes in the network, which is generally analyzed using centrality. The project attribute of nodes is used to divide nodes based on the number of items (i.e., projects) they participate in.

##### Structural Attributes of Nodes

Centrality is a measure of the importance of a node and its ability to collaborate with other nodes [42,43]. Therefore, we analyze the structural attributes of nodes in a multi-project KCN from two perspectives: strength centrality [44] and betweenness centrality [45].

(1) Strength centrality: In the multi-project KCN, the weight of connecting edges between nodes is obtained by combining the collaboration frequency of two nodes and the evaluation of the collaboration content. Therefore, we first determine the top ten nodes in the network in terms of the frequency, semantic weight, out-strength, and in-strength of the active collaboration of nodes, as shown in Table 2. The order of the top ten nodes is different for each indicator value, but the nodes are completely consistent. Further, Table 2 shows that nodes with professional knowledge are willing to actively seek collaboration after entering the community, and they also occupy a central position in the network. These nodes have greater influence in the community, and other nodes are willing to actively collaborate with them. If this willingness to collaborate decreases, the network performance will be affected to some extent.

(2) Betweenness centrality: Table 3 shows the top ten nodes in terms of the betweenness value in the network. There is a large difference between these nodes, and 55.6% of all nodes in the network have a betweenness value of 0. This indicates that only a few users occupy key collaborative positions in the network, making it important to identify them. Table 2 also shows that among the three indicators of betweenness centrality, out-strength, and in-strength, the coincidence degree of the top five nodes reached 80%, indicating that users who have strong collaboration abilities in the network also have important connection bridges.

##### Project Attributes of Nodes

**Figure 3:**
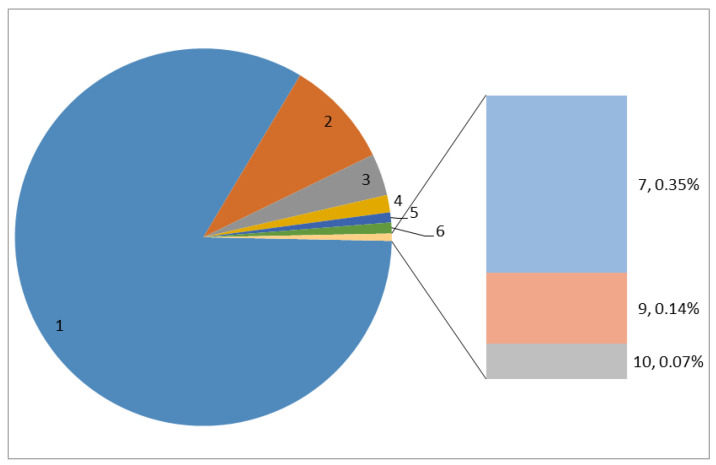
Distribution of the number of projects users participated in.

As previously stated, nodes have the autonomy to choose to participate in one or more projects in the OSC. The more projects that users participate in, the more satisfied they are with the project settings and the more loyal they are to the community. Figure 3 shows the number of projects that users participated in. We see that the majority of users (83.26%) collaborated on only one project in the community. Further, the users that participated in multiple projects account for only 16.74% (only 0.56% of the users participated in seven or more projects), and the collective out-strength of these nodes accounted for 56.32% of the total. Among those nodes who participated in the collaboration of only a single project, some have high betweenness values and occupy key positions in the network. Therefore, to explore the importance of nodes that participated in multiple projects and nodes that participated in single projects in the OSC, we first divide them according to the number of projects they participated in (m). If m≥2, these nodes are called multi-project nodes, and if m=1, these nodes are called single-project nodes.

**Figure 4:**
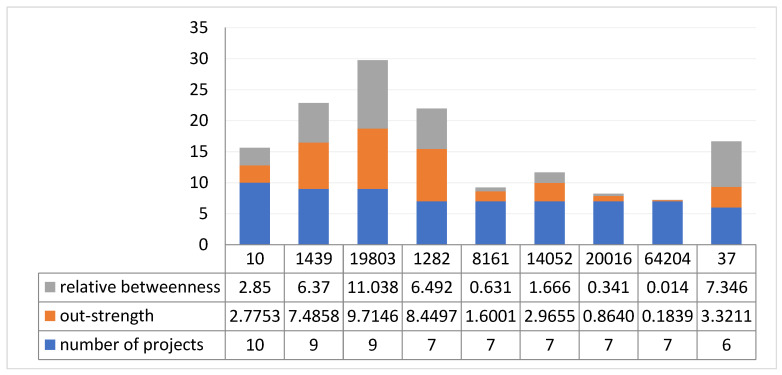
Out-strength and relative betweenness of top ten nodes (based on the number of projects they collaborated on).

It is a coarse-grained method to divide users by the number of projects they collaborated on, and many are divided to the same level. Further, through topological analysis, we find that the values of out-strength and betweenness of the nodes are different despite the nodes having collaborated on the same number of projects. This indicates that their roles and contributions to the community are different. Figure 4 shows the respective out-strength and relative betweenness of the ten nodes who participated in the most projects. Here, we see that the values of out-strength and relative betweenness of nodes who participated in more projects are lower than those who participated in fewer projects. This shows that in the multi-project KCN, it is still necessary to sort the importance of nodes according to their different topological attributes, to identify the characteristics of nodes with different roles and failure modes.

#### 2.2.3. Characteristics of Knowledge Collaboration Behavior between Nodes

Our analysis shows that users have different behaviors and positions in the community, including those who (1) actively contribute knowledge, (2) act as “intermediaries” to disseminate knowledge, and (3) are at the edge of browsing and searching.

To analyze edge connection attributes, we first divide knowledge collaboration behavior into our two primary areas of focus: knowledge contribution behavior and knowledge dissemination behavior. The edge weight of our KCN is weighted based on the strength of both the collaboration content and collaboration frequency, so it can effectively describe the knowledge collaboration behavior between nodes. If the order is based on the size of the edge weight, it can reflect the order of the knowledge collaboration behavior between nodes. In addition, the edge betweenness in the network is the number of times that the edge acts as the intermediary in the network, which is an important parameter used to measure knowledge dissemination behavior. If the order is based on the edge betweenness, it can reflect the strong and weak order of knowledge transmission between nodes. Table 3 shows the top ten edges in terms of weight and betweenness.

Table 3 shows that even among the top ten edges (in terms of weight and betweenness), the edge weights increased by 74.36% from the minimum to the maximum, and the edge betweenness increased by 64.56%. This shows that connections at the same node are different from one another: some are the main knowledge collaboration behavior and some are the main knowledge dissemination behavior. Further, their contributions to knowledge and the extent of knowledge dissemination are also different. Then, the willingness of nodes to cooperate decreases, and different connections have different effects on network performance.

## 3. Dynamic Robustness Analysis of Knowledge Collaboration Network

### 3.1. Robustness Evaluation Index

Network robustness can be defined as the degree of retention of network performance when network nodes or edges fail [46]. The impact of such failure for the KCN of an OSC includes (1) the destruction of network connectivity, which reduces the knowledge collaboration intensity, and (2) the decrease of network efficiency, which increases the difficulty of knowledge collaboration. As such, the robustness evaluation index proposed in this paper includes both network connectivity and weighted efficiency.

#### 3.1.1. Relative Size of Network Connectivity, S

To reflect the degree of network connectivity retention after the network is attacked, the relative network connectivity size, S, is defined as the relative size of the largest connected sub-graph node intensity of the network:

(2) $$S\;=\frac{S_{lc}^{\prime }}{S_{lc}}$$

where $S_{lc}^{\prime }$ is the sum of the node intensity of the maximum connected sub-graph of the network after being attacked, and $S_{lc}$ is the sum of the node intensity of the original network.

The calculation formula for the sum of the node intensity is

(3) $$S_{lc}=\sum_{j=1}^{N}w_{ij}$$

where N is the total number of nodes in the network and $w_{ij}$ is the edge weight of nodes i and j. In the weighted KCN of the OSC, node intensity represents the knowledge collaboration intensity. The smaller the value of S, the greater the decrease in knowledge collaboration intensity after the network is attacked (i.e., the lower the robustness of connectivity), and vice versa.

#### 3.1.2. Relative Size of Weighted Efficiency, *H*

Network efficiency describes the difficulty of information dissemination. It is expressed as the sum of the efficiency of all nodes, where node efficiency is the reciprocal of the shortest path between two nodes [47]. In the directed weighted network, the efficiency of knowledge collaboration, $E_{G}$, is expressed as

(4) $$E_{G}=\frac{1}{n\left(n-1\right)}\sum_{i\neq j}\frac{1}{{\left(d_{w}\right)}_{i,j}}$$

where the directed weighted shortest path, ${\left(d_{w}\right)}_{i,j}$, is the minimum sum of the weights necessary to travel from nodes i to j. To reflect the degree of knowledge collaboration efficiency retention after the network is attacked, the relative knowledge collaboration efficiency size, H, is defined as

(5) $$\;H\;=\;\frac{E_{G}{}^{\prime }}{E_{G}}$$

where $E_{G}{}^{\prime }$ is the weighted efficiency of the attacked network and $E_{G}$ is the weighted efficiency of the original network. The value range of H is $\left[0,\;1\right]$. When H=0, network efficiency drops to its lowest after the attack, that is, designers in the network do not have any form of collaboration. When H=1, the efficiency of the whole network remains at the original level, where the failure of edge weights has no impact on network efficiency.

### 3.2. Failure Mode Design

The design of failure modes is key to robustness analysis. We design two failure modes to study the robustness of the multi-project KCN: (1) node failure, where user resources are lost due to the impact of both the external environment and the internal ecology; and (2) edge failure, where the degradation of knowledge collaboration behavior is caused by a reduction in the willingness of users to participate in collaboration and the decline of collaboration strength.

#### 3.2.1. Node Failure

The node failure mode represents the performance of a node exiting the project or ceasing collaboration. Nodes can be categorized in two ways. First, by those that participate in multi-project collaboration and those that participate only in single-project collaboration. Second, by those that have high out-strength and actively participate in knowledge collaboration (known as knowledge collaboration nodes) and those that have high betweenness and act as important “bridge” nodes in the network, maintaining information transmission distance and knowledge collaboration efficiency (known as knowledge dissemination nodes). As such, the nodes can be classified as either (1) multi-project knowledge collaboration nodes, (2) multi-project knowledge dissemination nodes, (3) single-project knowledge collaboration nodes or (4) single-project knowledge dissemination nodes. The failure modes and calculation process of the above four nodes are shown in Table 4.

#### 3.2.2. Edge Failure

The edge failure mode is a manifestation of the degradation of a node’s willingness to collaborate in the OSC. Research shows that a good collaboration mechanism, mutual benefit mechanism, and enterprise incentive mechanism are important to promote the sustainable collaboration of community members [41]. The rationality of these mechanisms will determine the willingness of users to participate in knowledge innovation and collaboration in the OSC. If the community does not (a) formulate corresponding incentive measures for different users, (b) establish a good collaborative behavior agreement and coordination mechanism, and (c) clearly determine the intellectual property, a user’s willingness to collaborate will decrease. Using the same criteria as with the node division stated in Section 3.2.1, four edge failure modes are designed. These failure modes and their respective calculation processes are shown in Table 5.

## 4. Multi-Project Network Robustness Simulation Experiment

### 4.1. Robustness Simulation Test and Result Analysis under Node Failure Mode

**Figure 5:**
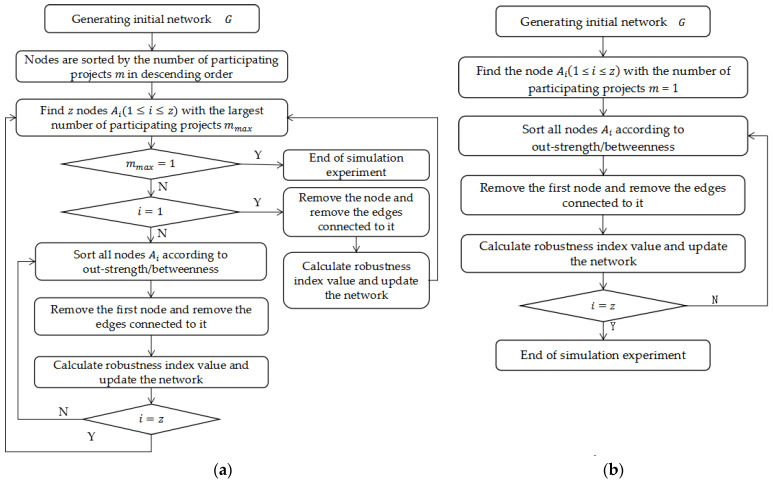
Node failure flow chart for (**a**) multiple-project nodes and (**b**) single-project nodes.

**Figure 6:**
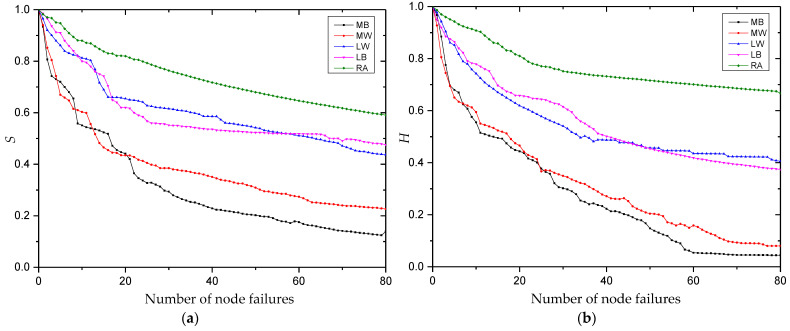
Robustness index values for (**a**) the relative size of network connectivity, S, and (**b**) the relative size of weighted efficiency, H.

The simulation process for the node failure mode is shown in Figure 5. This starts from the final developmental stage of the multi-project network, and it simulates the changes in robustness indicators during the node failure process. We use Python 3.7 programming to simulate the change process of the index value after the network faces the failure of different nodes, and we use Origin Pro 9.0 software to create a comparison chart of the experimental results. To compare the change of network robustness under different failure modes, the first 80 simulation results are taken for comparative analysis, where Figure 6a shows the change in the relative size of network connectivity under the node failure mode and Figure 6b shows the change of the relative size of knowledge collaboration efficiency.

Figure 5 shows that the robustness index values of the multi-project KCN show a downward trend when nodes fail successively. This decline rate is fastest when multi-project nodes (i.e., MW or MB) fail successively, followed by single-project nodes (i.e., LW or LB), then random nodes (i.e., RA).

A paired T-test is conducted on the index values under the five failure modes, as shown in Table 6. The index values are significantly different for each failure mode. Regarding robustness: RA > LB > LW > MW > MB.

Figure 6 shows that with the failure of nodes, the decline rate of indicator values is the smallest under successive random node failure (RA). When the failure index values of multi-project nodes decline by 90% in succession, random failure only decreases by 30%. This is due to the large-scale, loose state of the multi-project KCN. Most nodes are at the edge, and the probability of random loss is high. Their failure causes less damage to the network than the four other types of failure (i.e., LB, LW, MW and MB), which demonstrates that the network has a high robustness to the random failure of nodes. In contrast, the robustness is low both for the failure of single-project nodes and particularly for the failure of multi-project nodes.

Figure 6 also shows that when 10 nodes in the network fail, the relative connectivity size decreases by 50% for the MB failure mode, 45.36% for the MW failure mode, 30.8% for the LB failure mode and 26.58% for the LW failure mode. In addition, the relative efficiency of knowledge collaboration decreases by 47.69% for the MB failure mode, 50.3% for the MW failure mode, 20.2% for the LB failure mode and 18.96% for the LW failure mode. When 20 multi-project nodes are removed, the relative sizes of network connectivity and knowledge collaboration efficiency decrease by 60%, while the same degree of decline requires the failure of 80 single-project nodes. This shows that most multi-project nodes are in the center of the network, and they have contact with more users than single-project nodes. When multi-project nodes leave the community, they quickly disperse the network, dividing it from a huge, connected graph to isolated sub-graphs.

Based on the node attributes of the multi-project KCN, nodes with a higher betweenness and out-strength in the initial network have a higher overlap rate, so the changes in initial index values of the node failure simulation are more consistent. The continuous failure of knowledge dissemination nodes is more harmful to the network than the continuous failure of knowledge contribution nodes (i.e., the network shows lower robustness). In the early stage of the simulation experiment, the declining rule of the index value presents as a ladder shape. This is because node division in the failure mode setting was first based on the number of projects that the nodes participated in (as stated in Table 4). Many nodes were sorted to the same level, so the betweenness and out-strength of nodes in the same level show large differences. However, only a small number of single-project nodes have a high betweenness and out-strength, so the index value decreases greatly in the early stage of failure simulation. The subsequently failed nodes are all from the outer layer of the network, and their failure has progressively less impact on network performance. As such, the decline of the index value also progressively decreases.

### 4.2. Robustness Simulation Experiment and Result Analysis of Edge Failure Mode

**Figure 7:**
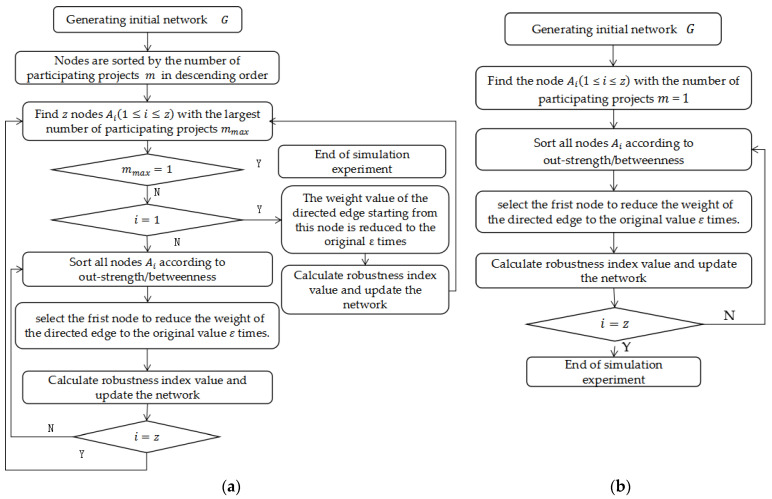
Edge failure flow chart for (**a**) multi-project nodes and (**b**) single-project nodes.

**Figure 8:**
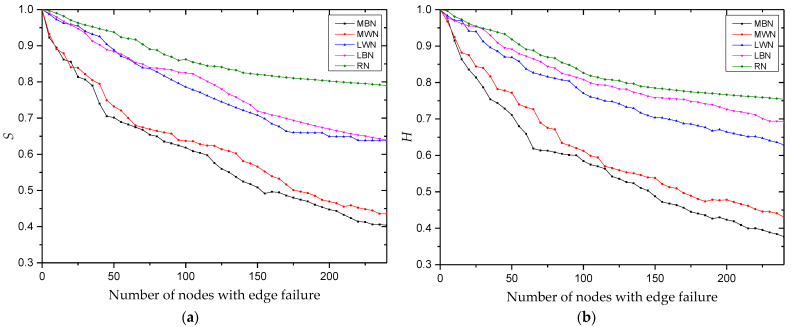
Robustness index values for (**a**) the relative size of network connectivity, S, and (**b**) the relative size of weighted efficiency, H.

Based on the edge failure modes constructed in Section 3.2.2, Figure 7 shows a simulation flow chart for (a) edge failure for multi-project nodes and (b) edge failure for single-project nodes. To more clearly observe the impact of edge failure on robustness, the degradation coefficient ε=0.8 is taken. We use Python 3.7 to program and simulate the index changes of multi-project network robustness when the network faces edge failure. As 241 nodes participate in multi-project collaboration in the KCN, the simulation process runs 240 times. We then use Origin Pro 9.0 to draw the experimental results for (a) the trend of the relative size of network connectivity and (b) the trend of the relative size of knowledge collaboration efficiency, as shown in Figure 8.

The number of simulations is limited because of the small number of multi-project nodes in the community. However, Figure 8 still shows that when the edge failure of multi-project nodes (i.e., MWN or MBN) occurs, the decrease of the index value is significantly larger than that of the edge failure of single-project nodes (i.e., LWN or LBN). This is because multi-project nodes have a higher strength and are more connected, that is, they have more edges through which to collaborate, so the failure of these nodes is more destructive to the network. The decrease of the edge failure index value of random nodes (i.e., RN) is the smallest. Further, for multi-project nodes, the edge failure of knowledge dissemination nodes (i.e., MBN) is more destructive to the network than the edge failure of knowledge collaboration nodes (i.e., MWN), whereas, for single-project nodes, the edge failure of knowledge dissemination nodes (i.e., LBN) is less destructive to the network than the edge failure of knowledge collaboration nodes (i.e., LWN).

A paired T-test is conducted on the index values under the five failure modes, as shown in Table 7. The index values are significantly different for each failure mode. Regarding robustness: RN > MBN > MWN > LWN > LBN.

Figure 8 shows that although the number of multi-project nodes is limited, when their edges fail, network connectivity and knowledge collaboration efficiency decrease significantly (i.e., by 60%). In contrast, failure of the same number of edges of single-project nodes reduces these values by only 30%. Among the multi-project nodes, the degree of damage to the network is slightly higher for the edge failure of knowledge collaboration nodes than for the edge failure of knowledge dissemination nodes. This result has also been verified many times in other research literature [26].

For the multi-project nodes, edge failure of knowledge dissemination nodes has a large impact on network performance. For the edge failure of single-project nodes, the opposite is true (the edge failure of knowledge collaboration nodes is more destructive to the network than knowledge dissemination nodes). This is because most single-project nodes occupy non-core positions in the network, so their edge failures only destroy the collaboration between the outer nodes of the network, which causes limited damage to the network. By comparing the network robustness indicators under the node failure mode with those under the edge failure mode, we find that the robustness of the network under edge failure is significantly higher than under node failure. For example, when 20 multi-project nodes fail, the network connectivity and knowledge collaboration efficiency decline by 60%, while the same percentage of decline requires the edge failure of 240 nodes. This is because when a node in the network fails, all collaborative relationships connected to the node are destroyed. In contrast, it is difficult to destroy the network structure through edge failure alone because such a failure only reduces the strength of the collaboration relationship between two nodes. Finally, when 150 multi-project knowledge dissemination nodes fail, the network connectivity and knowledge collaboration efficiency decrease by 50% and 53.6%, respectively, whereas for 150 multi-project knowledge collaboration nodes, they decrease by 45% and 47.3%, respectively. This shows that in the loose multi-project KCN, although the number of core nodes is small, they are critical to the network. When they leave the community or their willingness to collaborate decreases, it brings serious harm to the network.

## 5. Conclusions

This paper analyzes the characteristics of a multi-project KCN in the OSC, designs specific failure modes, and conducts robustness research. The following conclusions are drawn:

(1) The robustness of the multi-project network has the following characteristics: (a) low robustness in the case of deliberate failure, and high robustness in the case of random failure; (b) low robustness in the case of a node failure, and high robustness in the case of edge failure; and (c) low robustness in the case of a failure of multi-project nodes (or their edges), and high robustness in the case of a failure of single-project nodes (or their edges).
(2) For the node failure mode, the failure of multi-project nodes leads to the quick decline of network performance and later the collapse of the network. In contrast, the failure of single-project nodes leads to a slow decline in network performance, yet the network will still eventually collapse. For either scenario, the network is more sensitive to the failure of knowledge dissemination nodes than knowledge collaboration nodes.
(3) For the edge failure mode, the network has a low robustness to the failure of the edges of multi-project nodes and a high robustness to the failure of the edges of single-project nodes. For the multi-project nodes, the edge failure of knowledge dissemination nodes causes more damage to the network than the edge failure of knowledge collaboration nodes. For single-project nodes, the edge failure of knowledge collaboration nodes cause more damage to the network than the edge failure of knowledge dissemination nodes.

According to the analysis results, the following management recommendations can be obtained:

(1) Community managers should optimize their community’s ecology and create a good knowledge collaboration environment. The failure of nodes will quickly destroy the structure and performance of the network. Therefore, the primary concerns should be to optimize the community’s environment, improve the community’s competitiveness, effectively prevent the loss of users and improve users’ willingness to share knowledge [48]. Managers can start by (a) building a safe and stable community environment with clear intellectual property rights, (b) setting up reasonable incentive measures and (c) improving the conversion rate of products. In addition, essential tasks in the daily management and maintenance of the community include optimizing the collaboration platform, and making users have a good use of the community platform.
(2) Community managers should promote multi-project collaboration. Most multi-project nodes occupy a central position in the network. These nodes play a very important role in network connectivity and knowledge collaboration efficiency, and their continuous failure will completely destroy the robustness of the network, particularly if they are knowledge dissemination nodes. Therefore, managers should (a) strengthen the protection of multi-project users, (b) encourage such users to actively participate in collaboration, (c) improve the quality of knowledge collaboration, and (d) quickly spread any collaborated knowledge throughout the network. In addition, research shows that most users are willing to participate in multi-project collaboration [31]. Therefore, managers should encourage single-project users to collaborate on other projects by continuously optimizing the community ecology, releasing more attractive and innovative projects, creating more training opportunities, and setting reasonable project task modules.

## Figures and Tables

**Table 1:** Network topology parameters and structural characteristics of the multi-project KCN.

Network Size		Topological Parameters				Structural Characteristics		
Nodes	Edges	Average Out-Strength	Density	Average Path Length	Clustering Coefficient	Small-World Characteristic	Scale-Free Property	Assortativity
1410	18,469	0.119	0.0001	3.105	0.097	Yes	Yes	No

Note: According to Davis, Yoo and Baker [40], the small-world parameters can be expressed as $\mathrm{SW}=\left[Cactual/Lactual]*[Lrandom/Crandom\right]$, where Cactual is the average clustering coefficient of the network, Lactual is the average path length, $Lrandom=ln\left(n\right)/ln\left(‹k›\right),\;Crandom=‹k›/n,\;n$ is the number of nodes and *‹k›* is the average degree. The network efficiency is $E=\frac{1}{n\left(n-1\right)}\sum_{i\neq j}\frac{1}{d_{ij}}$, $\mathrm{where}\;d_{ij}$ is the length of the weighted shortest path from node i to j [41].

**Table 2:** Top ten nodes in terms of frequency, semantic weight, out-strength, in-strength, and betweenness.

Node	Frequency	Node	Semantic Weight	Node	Out- Strength	Node	In- Strength	Node	Betweenness
19,803	981	1282	2064.25	19,803	9.715	19,803	10.137	19,803	218,981.32
1439	841	19,803	2041.85	1282	8.449	37	5.321	37	145,842.98
1282	749	1347	1385.80	1439	7.486	1282	5.269	1282	128,908.91
1347	598	1439	1338.17	1347	6.210	1439	3.669	1439	126,908.63
52	563	52	846.88	52	4.911	1347	3.297	52	106,636.11
14,052	365	407	747.14	37	3.321	14,052	3.274	1347	91,292.38
37	362	37	624.39	407	3.249	52	2.664	10	56,544.84
10	328	10	451.30	14,052	2.965	198	2.255	407	45,410.023
407	305	14,052	442.07	10	2.775	1	2.186	14,052	33,045.941
198	301	198	439.51	9606	2.525	407	1.996	9606	24,465.762

**Table 3:** Top ten edges in terms of weight and betweenness.

	Edge	Weight	Edge	Betweenness
1	19803–1282	2	59581–19803	19005.7
2	1282–19803	1.6504	37–19785	16021.19
3	19803–49253	0.9645	1–10	14437
4	49253–1282	0.7702	11–52	9265.211
5	37–1282	0.6407	37–1439	8151.022
6	62210–19803	0.6252	37–1347	7816.247
7	14052–6	0.6147	1282–19803	7495.167
8	3606–1439	0.5746	10–3134	6752.929
9	1–1	0.5518	19803–52	6735.993
10	14052–10	0.5128	19803–1347	6569.205

**Table 4:** Node Failure Modes.

	Failure Mode	Failure Process
Node Failure Mode	Failure of multi-project knowledge collaboration nodes (MW)	(1) Multi-project nodes are ranked according to the number of projects they participate in, where nodes with the same number are also ranked according to their out-strength. (2) The hihighest-rankedode and its connected edges are removed. This is repeated n times to simulate successive node failure.
	Failure of multi-project knowledge dissemination nodes (MB)	(1) Multi-project nodes are ranked according to the number of projects they participate in, where nodes with the same number are also ranked according to their betweenness. (2) The highest-ranked node and its connected edges are removed. This is repeated n times to simulate successive node failure.
	Failure of single-project knowledge collaboration nodes (LW)	(1) Single-project nodes are ranked according to their out-strength. (2) The highest ranked node and its connected edges are removed. This is repeated n times to simulate successive node failure.
	Failure of single-project knowledge dissemination nodes (LB)	(1) Single-project nodes are ranked according to their betweenness. (2) The highest-ranked node and its connected edges are removed. This is repeated n times to simulate successive node failure.
Random failure	Random failure of nodes (RA)	A random node and its connected edges are removed. This is repeated n times to simulate the irregular failure of users.

**Table 5:** Edge Failure Modes.

	Failure Mode	Failure Process
Edge Failure Mode	Edge failure of multi-project knowledge collaboration nodes (MWN)	Multi-project nodes are ranked according to the number of projects they participate in, where nodes with the same number are also ranked according to their out-strength. (2) Reduce the weight of directed edges (point from the highest ranked node to other nodes) to the original value *ε* times. This process is repeated n times to simulate successive failure.
	Edge failure of multi-project knowledge dissemination nodes (MBN)	(1) Multi-project nodes are ranked according to the number of projects they participate in, where nodes with the same number are also ranked according to their betweenness. (2) Reduce the weight of directed edges (point from the highest ranked node to other nodes) to the original value *ε* times. This process is repeated n times to simulate successive failure.
	Edge failure of single-project knowledge collaboration nodes (LWN)	Single-project nodes are ranked according to their out-strength. (2) Reduce the weight of directed edges (point from the highest ranked node to other nodes) to the original value *ε* times. This process is repeated *n* times to simulate successive failure.
	Edge failure of single-project knowledge dissemination nodes (LBN)	(1) Single-project nodes are ranked according to their betweenness. (2) Reduce the weight of directed edges (point from the highest ranked node to other nodes) to the original value *ε* times. This process is repeated *n* times to simulate successive failure.
Random failure	Random failure of edges (RN)	Reduce the weight of directed edges (point from the random node to other nodes) to the original value *ε* times. This is repeated *n* times to simulate successive failure.

**Table 6:** Paired sample T-test under different failure modes (*α* = 0.05).

	Failure Mode	M	SD	95% Confidence		t	df	Sig
				Lower Limits	Upper Limits			
*H*	RA-LW	0.20588	0.008792	0.188384	0.223378	23.416	80	0.000
	LW-LB	0.01042	0.004013	0.002433	0.018408	2.596	80	0.011
	LB-MW	0.16216	0.006394	0.149192	0.175128	25.360	36	0.000
	MW-MB	0.01422	0.005219	0.003592	0.024855	2.725	32	0.010
*S*	RA-LW	0.131216	0.034775	0.123527	0.138906	33.960	80	0.000
	LW-LB	0.008251	0.036572	0.000164	0.016337	2.031	80	0.046
	LB-MW	0.186069	0.044733	0.173615	0.198523	29.995	51	0.000
	MW-MB	0.021539	0.009019	0.003143	0.039934	2.388	31	0.023

**Table 7:** Paired sample T-test under different edge failure modes (*α* = 0.05).

	Failure Mode	M	SD	95% Confidence		t	df	Sig
				Lower Limits	Upper Limits			
*H*	RN-LBN	0.048859	0.003368	0.042156	0.055563	14.504	50	0.000
	LBN-LWN	0.058749	0.003126	0.052528	0.064970	18.793	50	0.000
	LWN-MWN	0.223773	0.008269	0.207219	0.240326	27.059	50	0.000
	MWN-MBN	0.044454	0.003763	0.036883	0.052024	11.813	50	0.010
*S*	RN-LBN	0.122428	0.006578	0.109335	0.135520	18.609	50	0.000
	LBN-LWN	0.003362	0.002658	−0.00192	0.008652	1.265	50	0.021
	LWN-MWN	0.165131	0.007121	0.150908	0.179354	23.187	50	0.000
	MWN-MBN	0.021268	0.002186	0.016901	0.025635	9.726	50	0.000

## Data Availability

The used and analyzed datasets during the present study are available from the corresponding author upon reasonable request.

## Abbreviations

The following abbreviations are used in this manuscript:

KCN	Knowledge Collaborative Network
OSC	Open source communitiy
OSP	Open source project
MW	Failure of multi-project knowledge collaboration nodes
MB	Failure of multi-project knowledge dissemination nodes
LW	Failure of single-project knowledge collaboration nodes
LB	Failure of single-project knowledge dissemination nodes
RA	Random failure of nodes
MEN	Edge failure of multi-project knowledge collaboration nodes
MBN	Edge failure of multi-project knowledge dissemination nodes
LWN	Edge failure of single-project knowledge collaboration nodes
LBN	Edge failure of single-project knowledge dissemination nodes
RN	Random failure of edges
